# Diversity and Dynamics of Seaweed Associated Microbial Communities Inhabiting the Lagoon of Venice

**DOI:** 10.3390/microorganisms8111657

**Published:** 2020-10-26

**Authors:** Abdul-Salam Juhmani, Alessandro Vezzi, Mohammad Wahsha, Alessandro Buosi, Fabio De Pascale, Riccardo Schiavon, Adriano Sfriso

**Affiliations:** 1 Department of Environmental Sciences, Informatics and Statistics, Ca’Foscari University of Venice, Via Torino 155, 30172 Venezia, Italy; alessandro.buosi@unive.it (A.B.); sfrisoad@unive.it (A.S.); 2 Department of Biology, University of Padua, Via U. Bassi 58/B, 35131 Padua, Italy; alessandro.vezzi@unipd.it (A.V.); fabulo84@gmail.com (F.D.P.); riccardo.schiavon@gmail.com (R.S.); 3 Marine Science Station, The University of Jordan, Aqaba branch, 77110 Aqaba, Jordan; m.wahsha@ju.edu.jo; 4 BMR Genomics, Via Redipuglia 21a, 35131 Padua, Italy

**Keywords:** bacterial community, seaweeds, Illumina, lagoon of Venice, anthropogenic stressors

## Abstract

Seaweeds are a group of essential photosynthetic organisms that harbor a rich diversity of associated microbial communities with substantial functions related to host health and defense. Environmental and anthropogenic stressors may disrupt the microbial communities and their metabolic activity, leading to host physiological alterations that negatively affect seaweeds’ performance and survival. Here, the bacterial communities associated with one of the most common seaweed, *Ulva laetevirens* Areshough, were sampled over a year at three sites of the lagoon of Venice affected by different environmental and anthropogenic stressors. Bacterial communities were characterized through Illumina sequencing of the V4 hypervariable region of 16S rRNA genes. The study demonstrated that the seaweed associated bacterial communities at sites impacted by environmental stressors were host-specific and differed significantly from the less affected site. Furthermore, these communities were significantly distinct from those of the surrounding seawater. The bacterial communities’ composition was significantly correlated with environmental parameters (nutrient concentrations, dissolved oxygen saturation, and pH) across sites. This study showed that several more abundant bacteria on *U. laetevirens* at stressed sites belonged to taxa related to the host response to the stressors. Overall, environmental parameters and anthropogenic stressors were shown to substantially affect seaweed associated bacterial communities, which reflect the host response to environmental variations.

## 1. Introduction

The surface of seaweeds represents a highly active interface between the host and the surrounding environment. This interface is involved in exchange processes such as the uptake and release of nutrients, waste products, and secondary metabolites [1]. Moreover, the seaweed surface provides a suitable substratum for the settlement of different microorganisms and secretes various organic substances that promote bacterial division and the formation of microbial biofilms [2,3]. The symbiotic interactions between seaweeds and bacteria are mainly influenced by environmental parameters, such as the availability of inorganic nutrients and organic matter [4]. The diverse associated bacterial communities can also aid the survival of the holobiont (host plus its symbionts), allowing the host to cope with rapid and severe environmental changes [5]. Some bacterial strains associated with seaweeds are suggested to play a role in stress tolerance [6,7], and bioremediation of contaminants, including hydrocarbons and fertilizers [8]. 

Despite the fundamental role of seaweed associated microbial communities (SAMCs) on the hosts’ health, there is little information on how these communities vary at large spatial scales and what are the main drivers of this variation [1,9,10]. The characterization of SAMCs through next-generation sequencing (NGS) provides a holistic assessment of bacterial community structure and diversity, which has lately emerged as a powerful tool to examine bacterial communities associated with marine eukaryotes. Consequently, different recent researches have analyzed the dynamics and spatio-temporal variations of SAMCs [6,11,12,13]. 

Studies on SAMCs suggested that bacterial assemblages were distinct from the surrounding environment and largely host-specific [14]. Moreover, SAMCs displayed considerable spatial and temporal variation [15]. Recent investigations demonstrated that environmental and anthropogenic pressures could disrupt SAMCs. The microbial communities associated with the brown seaweed *Ecklonia radiata* (C. Agardh) J. Agardh were strongly influenced by stress related to coastal urbanization [16]. Furthermore, the bacterial communities associated with the red seaweed *Asparagopsis* were found to have host-specificity and were modulated by environmental conditions. Still, it remains unclear whether this environmental effect reflects the host’s selective requirements or the locally available bacteria [17]. Despite the increased interest in seaweed bacterial associations, the host seaweed species’ functional relationships remain largely unknown. Understanding the dynamics of SAMCs would allow one to extensively elucidate the overlooked mechanisms behind algal responses to environmental or anthropogenic stressors. 

Seaweeds of the family Ulvaceae represent one of the most important primary producers of coastal marine ecosystems. *Ulva* represents an important natural resource, providing food—for grazer’s marine animals, including crustaceans, such as amphipods and mollusks [18]. During mild eutrophic phenomena, the more significant presence of nutrients in seawater favors the increase of these nuisance seaweeds, which consume the excess of nutrients. The analysis of environmental data in the lagoon of Venice (after that called LV) showed that despite the preferential existence of several *Ulva* species in eutrophic environments, *U. laetevirens* was more abundant in areas where nutrient levels (mostly, phosphate and ammonium) and phytoplankton were low [18]. The nutrient surplus and anthropogenic pollution depressed the phytoplankton growth in the LV during the last decades [19]. In fact, the excess of inorganic nutrients in the water column triggered a series of algal blooms. Tionitrophilous opportunistic species like *U. laetevirens* and *U. rigida*, have become the dominant primary producers and outcompeting phytoplankton and favoring cyanobacteria’s blooms during their seasonal collapse [20].

This study investigated the bacterial communities associated with *U. laetevirens* in three sites across the LV. *Ulva* is an opportunistic species with high adaptive potential that utilizes the excess of nitrogen in the environment and replaces the other seaweed species. This species represents an excellent model to study holobiont adaptation to different environments and anthropogenic impacts. The shift and dynamics of the bacterial communities during an *Ulva* growth season in areas affected by various anthropogenic stressors were characterized using 16S rRNA gene barcoding through next-generation sequencing. Besides, SAMCs of each site were compared with the microbial communities of surrounding seawater. We expect that host-associated bacterial communities will cluster differently in response to the anthropogenic stressors, a variety of environmental parameters, and seasonal fluctuations. 

## 2. Materials and Methods 

### 2.1. Study Area 

**Figure 1:**
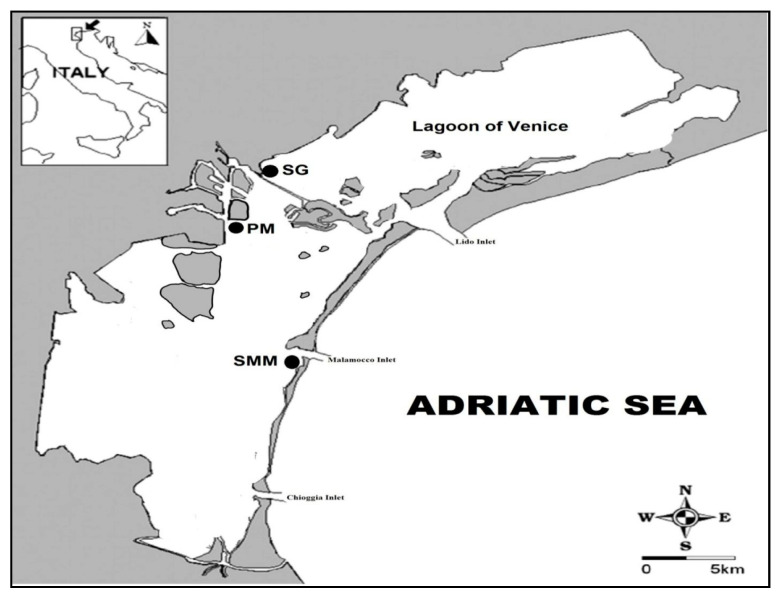
Map of the sampling sites across the central basin of the Lagoon of Venice (Italy). SMM: Santa Maria del Mare, PM: Porto Marghera, and SG: San Giuliano.

The LV is a complex, heterogeneous, and continuously evolving dynamic system, sensitive to an array of external drivers and pressures. Both natural and anthropogenic stressors significantly affect the lagoon ecosystem [21]. The lagoon is characterized by many sub-basins with different hydro-morphological conditions and habitats affected by different nutrient loadings, trophic status, and salinity, and is colonized with high biodiversity. According to the literature [22,23,24,25], three sampling sites were selected in the central basin (Figure 1). Santa Maria del Mare (SMM) is located close to the Malamocco inlet, which connects the lagoon with the northern Adriatic Sea and, therefore, experiences very effective tidal water exchanges. It is characterized by a high ecological status (high hydrodynamics, low nutrient concentrations, extensive seagrass meadows, and the absence of algal blooms). This area presents a low level of pollutants; hence, it is poorly impacted by anthropogenic pressures. Porto Marghera (PM) is close to the inner border of the lagoon, at the center of the NIS (National Interest Site) area, which is identified as an area of high environmental risk. PM is affected by the pollutants released from the petrochemical pole’s industrial activities and by urban and agricultural water inputs that discharge pollutants into the lagoon [26,27]. San Giuliano (SG) is close to the inner border of the lagoon; it has very poor water exchange and is characterized by hypertrophic conditions and marked industrial and urban contamination [28,29]. SG is influenced by the freshwater inputs of the Osellino River which is conveyed in the lagoon nutrients and pollutants [30]. It is also characterized by anoxic sediments, persistent water anoxia in spring-summer, and high variability of environmental parameters such as water transparency and salinity.

### 2.2. Water Sampling and Environmental Parameters Monitoring

Water samples were collected in four different seasons (winter (December) 2016, spring (April), summer (July), and autumn (September) 2017). At each site, pH values (accuracy ± 0.015 units) and redox potential (Eh) (accuracy ± 0.15%) were measured by a portable pH-meter (model PH25+) (CRISON instruments, Barcelona, Spain). Temperature and dissolved oxygen concentration (mg L^−1^) were measured by a portable oximeter (OXI45+) (CRISON instruments, Barcelona, Spain). The percentage of oxygen saturation was calculated by the formula of Weiss [31]. Six surface water samples were collected using a bucket and mixed in a tank. Sub-samples of 0.5 to 1.0 L, depending on water turbidity, were filtered through GF/F Whatman glass microfiber filters (porosity 0.7 μm). Filtered waters were stored frozen at −20 °C for nutrient (ammonium, nitrite, nitrate, reactive phosphorus, and silicate) determination according to the spectrophotometric analysis procedures reported by Strickland and Parson [32]. The quantification at different wavelengths of phosphates, silicates, and inorganic nitrogen compounds occurs by comparing calibration curves made with seawater. All colorimetric analyses were performed in triplicate. Differences in nutrient concentrations among sites were statistically evaluated (one-way ANOVA). Differences were considered significant where the *p*-value < 0.05. Chlorophyll-*a* (Chl-*a*) concentrations were measured by spectrophotometric analysis [33] using acetone (90%) extraction protocol. Twenty milliters of water sub-samples were collected to determine salinity by the chlorine titration method [34]. Two additional 500 mL water sub-samples were filtered in situ through GF/F Whatman glass microfiber filters (porosity 0.7 μm), previously dried at 110 °C for two hours, to obtain the concentration (mg L^−1^) of the suspended particulate matter (SPM), as described previously by Sfriso et al. [35]. 

### 2.3. Microbial Community Analyses

#### 2.3.1. Seaweed Sampling 

The samples of *U. laetevirens* were collected from each site to obtain the associated microbial communities. Specifically, several thalli of *U. laetevirens* fixed on the substratum were sampled from the intertidal zone. The selected thalli were washed with seawater to eliminate or reduce the attached invertebrates and settled sediment and preserved in bottles of pre-sterilized Artificial Sea Water (ASW) to avoid inappropriate contamination. The bottles were kept in ice until laboratory analysis. Two replicates per station were composed of 10 equally sized thalli of *U. laetevirens* were used for analysis. 

#### 2.3.2. DNA Extraction, Sequencing and 16S rRNA Sequence Data Processing

In the laboratory, samples were immediately subjected to smooth sonication (3 × 30 s, frequency: 35 KHz, Bendelin sonorex, Germany) to ensure bacterial cells’ detachment from *U. laetevirens* surface. The seaweed fragments were removed with sterile tweezers under sterilized conditions followed by filtration of the remaining ASW (including bacterial cells) with 0.20 µm nitrocellulose filters to trap bacterial cells. The filters for each replicate were placed in sterile tubes and washed with 10 mL of buffer solution (200 mM Tris-HCl pH 8, 10 mM EDTA, and 0.24% Triton X-100; [36]). The tubes were centrifuged at 5000 rpm for 20 min to obtain the bacterial pellets. Microbial DNA was extracted from the pellets using the DNeasy^®^ PowerSoil^®^ Kit (Qiagen, Germantown, MD, USA) following the manufacturer’s specifications. The integrity and quantity of DNA extracts were determined by agarose gel electrophoresis and Qubit Fluorometer 2.0 (Invitrogen, Waltham, MA, USA). DNA was stored at −20 °C for further use. The two replicates for each sample of extracted DNA were mixed to obtain a higher DNA quantity. For comparison purposes, total DNA was also extracted from seawater samples that surrounded the collected seaweeds following the same DNA extraction protocol during summer and autumn.

PCR amplicon libraries for Illumina NextSeq 500 sequencing platform were constructed, using bacterial primers 515F (5′-GTGYCAGCMGCCGCGGTAA-3′) and 806R (5′-GGACTACNVGGGTWTCTAAT-3) targeting V4 hyper-variable region of bacterial 16S rRNA genes [37]. PCR conditions were: 98 °C for 4 min; 25 cycles of 98 °C for 20 s, 57 °C for 30 s, followed by 72 °C for 30 s, with a final extension of 72 °C for 5 min. Phusion^®^ (New England Biolabs, Ipswich, MD, USA) high-fidelity DNA polymerase was used in the amplification reaction. The PCR products were purified using Agencourt Ampure XP beads (Agencourt Bioscience Corp., Beverly, MA, USA) and quantified using a Qubit system (Invitrogen, Waltham, MA, USA). The amplicons were tagged using the Nextera XT DNA sample preparation kit (Illumina Inc., San Diego, CA, USA) with a dual indexing strategy. The purified amplicons were subjected to a second PCR with 12 cycles to attach the indexes. Equimolar amounts of Ampure XP beads purified tagged amplicons were pooled and analyzed through the Illumina^®^ NextSeq 500 platform using the Nextseq^®^ 500/550 High-output Kit v2 (Illumina, Inc., San Diego, CA, USA), according to the manufacturer’s instructions. 

Sequence analysis was restricted only to forward reads as reverse reads failed to pass the quality check—base quality dropped too early for the reverse reads to be useful (data are not shown). The analysis was performed with the DADA2 pipeline [38] using the dada2 R-package (v. 1.8.0). DADA2 algorithm identifies amplicon sequence variants (ASV), allowing accurate high resolution of sample composition from amplicon sequence data. Within the same pipeline, before the ASV identification step, primers were removed, and forward reads were trimmed at 125 bp to eliminate low-quality bases. Taxonomy assignment of resulting ASVs was performed using dada2 R-package built-in classifier against the SILVA SSU database v. 128 [39] with a 50% confidence threshold. The sequences were classified from phylum to genus level. After classification, features assigned to chloroplasts or mitochondria, as well as eukaryotic and unassigned sequences, were excluded from the dataset. Feature counts were normalized computing relative abundances: raw counts were divided by total counts per sample and then multiplied to the median total count. After this normalization, only ASVs with abundance above 3 × 10^−5^ were retained. The resulting features were used for explorative characterization of the bacterial communities.

The 16S amplicon sequences generated for this study can be found in the sequence reads archive (SRA) at NCBI with the accession number PRJNA656714. Table S1 shows the proper correspondences between sample IDs used in the present article and sample ID and accession numbers at SRA.

### 2.4. Microbial Community Structure

Alpha diversity of the *U. laetevirens* associated bacterial communities was characterized in the three selected sites during the four sampling periods. Besides, the seaweed surrounding seawater bacterial community of summer and autumn was considered. To estimate alpha diversity, data sets were rarefied at the number of sequences of the sample with the least sequencing depth. The rarefied ASVs table was applied to calculate alpha diversity statistics, including Chao I richness estimates [40], Simpson and Shannon indices, and the observed number of ASVs. Both data set rarefaction and alpha diversity estimation were computed by the MicrobiomeAnalyst tool [41]. 

The visualization of the temporal and spatial variation of SAMCs (beta diversity) was displayed by unconstrained ordination plots using the principal coordinate analysis (PCoA) based on a Bray-Curtis [42] distance matrix calculated from square-root transformed ASVs abundance data. Permutation multivariate analysis of variance (PERMANOVA+) was performed using the “Adonis” function from the vegan R-package to test for differences among samples with the factors: type of sample (seaweeds vs. water), season, and sampling site. Samples were considered statistically different at *p*-values < 0.05 and r-values close to one [43]. Analyses were done using 9999 permutations of residuals under a reduced model. To identify ASVs specific and shared among defined sample groups, a Venn-diagram was constructed using Venny 2.0 [44]. A similarity percentage analysis (SIMPER) was performed in PRIMER v. 6 [45] to identify those classes that most characterized the composition of *U. laetevirens* associated bacteria community at each sampling period or that mostly contributed significantly to the observed differences. The cut-off value was restricted to 60%. The average relative abundance of bacterial families was employed to compare the differences between SAMCs and the surrounding water communities using analysis of variance (one–way ANOVA). Differences were considered significant at *p* < 0.05 and highly significant at *p* < 0.001. Prior to the analyses, the distribution of each variable (average relative abundance) was checked for normality and homogeneity of variance using the Welch test.

### 2.5. Linear Discriminant Analysis (LDA) Effect Size (LEfSe) Measurement 

To detect bacterial taxa with a significant differential abundance between the sampling sites and the sampling periods, linear discriminant analysis effect size (LEfSe) measurement [46] was created according to the web-based tool [47]. For LEfSe, Kruskal–Wallis test by ranks were performed to detect the features (ASVs) with significant abundance, followed by LDA to evaluate the effect size of each differentially abundant ASV. Values were considered significant at *p* < 0.05 for both statistical methods. ASVs with markedly increased effect size were defined as those with an LDA score (log_10_) > 3. LDA score represents the discriminant rank of the features ASVs with differential abundance within sampling periods or among sampling sites. 

### 2.6. Relationship between SAMCs and Environmental Parameters 

The relationship between *U. laetevirens* associated bacterial communities and environmental parameters was explored using distance-based redundancy analysis (db-RDA) by CANOCO (v. 5.0) software. The normalized relative abundance of ASVs and the measured environmental parameters were used as species input and environmental input, respectively.

## 3. Results

### 3.1. Environmental Parameters Analyses

The seasonal measurements of the environmental physico-chemical parameters determined in the water column (pH, redox potential (Eh), temperature (Temp.), dissolved oxygen saturation (%DO), and salinity (Sal.)) in the stations of LV are shown in Table 1. The results of the sampling periods were significantly different (one-way ANOVA) for pH values (*p* < 0.016), %DO (*p* < 0.025), and water temperature (*p* < 0.01). The highest average value of pH was recorded at SMM. Redox potential (Eh) exhibited high variability among seasons where the lowest values were recorded in spring for all sites. Water temperature generally increased from the sea inlet (SMM) to the mainland (PM and SG); the highest seasonal variation was recorded at SG (14.0 to 29.8 °C). The highest %DO fluctuations were found at SG with a peak in spring (180%). Salinity also fluctuated among sites and seasons; the lowest value (6.40 psu) was measured at SG during winter whereas the highest one (32.5 psu) was recorded in summer at SMM. At SMM and PM, the average Chl-*a* concentrations were 1.04 and 1.92 µg/L, respectively, whereas, at SG the mean Chl-a concentration was 9.34 µg/L, mostly due to the spring bloom (29.9 µg/L). Chl-*a* did not exhibit a specific seasonal trend among sites.

**Figure 2:**
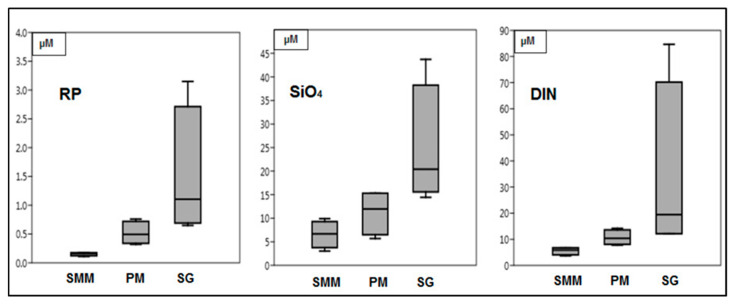
Concentrations of reactive phosphorus (RP), silicates (SiO_4_), and dissolved inorganic nitrogen (DIN) (µM) in the water column of LV. SMM: Santa Maria del Mare, PM: Porto Marghera, and SG: San Giuliano.

During the sampling periods, the sites differed significantly (one-way ANOVA) for the concentration of reactive phosphorous (RP) (*p* < 0.01), silicates (SiO_4_) (*p* < 0.01), and DIN (*p* < 0.018). The eutrophic conditions recorded at SG (RP: 0.7 to 3.2 µM, DIN: 12 to 85 µM, and SiO_4_: 14 to 44 µM), were explained by the heavy nutrient loading in this area (Figure 2). In the other two sites (SMM and PM), RP showed very low concentrations and seasonal changes (mean values: 0.15 and 0.52 μM at SMM and PM, respectively).

The lowest DIN concentrations were recorded at SMM in all the sampling periods. In contrast, the highest DIN concentration was found at SG in winter, mainly due to ammonium which contributed to ca. 70% of the total value. Conversely, high nitrate concentrations (until 63% of DIN) were recorded in winter and autumn at SMM and PM (Table S2). Silicates were generally higher in winter and reached the lowest spring and autumn values in all sampling sites.

### 3.2. Overview of Sequencing Output

In total 4.7 M raw reads were obtained but only forward reads were analyzed for a total of 2,253,643 reads. The DADA2 procedure identified 6935 ASVs; of these 19.4 and 8.7% were assigned to chloroplasts and mitochondria, respectively. Classification of ASVs against the Silva database resulted in 56.3% of the ASVs being classified at genus levels. Once sequences assigned to chloroplasts and mitochondria were removed and abundance normalization was applied, the average number of usable reads per sample became 102,090 (SD = 2313; min = 97,846; max = 105,691), and the total number of retained ASVs was 2307. Rarefaction curves showed saturation for all samples, indicating good diversity coverage (Figure S1).

### 3.3. Microbial Communities’ Structure

Fifteen bacterial phyla were identified in the *U. laetevirens* associated bacterial communities of LV sampling sites during the four sampling periods (Figure S3 and Table S3). The bacterial communities were predominantly composed of Proteobacteria and Bacteroidetes, which comprised more than 88% of the total sequences among all sites. The relative abundance of each phylum varied between sampling periods. For instance, the abundance of Cyanobacteria increased during summer in all the investigated sites (1.3, 8.0, and 2.6% of the total sequences at SMM, PM, and SG, respectively). Moreover, Planctomycetes increased significantly during spring at SG (11.8% of the total sequences) and Verrucomicrobia at SMM during the same season (18% of the total sequences). The eight less abundant phyla (Acidobacteria, Epsilonbacteraeota, Fusobacteria, Deinococcus-Thermus, Chloroflexi, Thaumarchaeota, Tenericutes, and Kiritimatiellaeota) accounted for ca. 2% of the total sequences only.

At the class level, the relative abundance of bacterial taxa varied during the sampling periods among all sites (Figure S3 and Table S3). Three classes dominated the seaweed surface: Alphaproteobacteria (phylum Proteobacteria), Bacteroidia (phylum Bacteroidetes), and Gammaproteobacteria (phylum Proteobacteria) which accounted for about 29, 21, and 37% of the total sequences, respectively. The remaining 21 classes accounted for more than 10% of the total sequences considering all samples. Also, the distribution of classes in the same sites changed during different sampling periods. For instance, Verrucomicrobiae (phylum Verrucomicrobia) relative abundance was higher at SMM during spring, whereas Oxyphotobacteria (phylum Cyanobacteria) class was relatively higher during summer at PM.

The SIMPER analysis revealed low dissimilarity (22.4%) between SMM and PM. The Gammaproteobacteria, Planctomycetacia, Alphaproteobacteria, Bacteroidia, Oxyphotobacteria, and Verrucomicrobiae bacterial classes contributed >81% of the observed difference. Lower dissimilarity (16.8%) was shown between SMM and SG where the same classes contributed to >79.0% of the difference. Likewise, the dissimilarity between PM and SG was 19.4%, where the classes Alphaproteobacteria, Gammaproteobacteria, Oxyphotobacteria, Planctomycetacia, and Bacteroidia contributed to 70.0% of the differences.

At a finer level of phylogenic resolution, the bacterial communities across sampling sites were more distinct. A subset of 240 seaweed associated ASVs (around 10.4% of the total ones) represented 58% of the overall diversity observed for all sites in all sampling periods. The distribution and abundance percentage of the most abundant ASVs varied between sites (Table 2). As a result, bacteria from the families Hyphomonadaceae (ASV9 and ASV12) and Nitrincolaceae (ASV1, ASV10, and ASV13) were remarkably abundant at PM. In contrast, the bacteria related to the families Flavobacteriaceae (ASV5, ASV7, and ASV27) and Rubritaleaceae (ASV21) were abundantly present at SMM.

**Figure 3:**
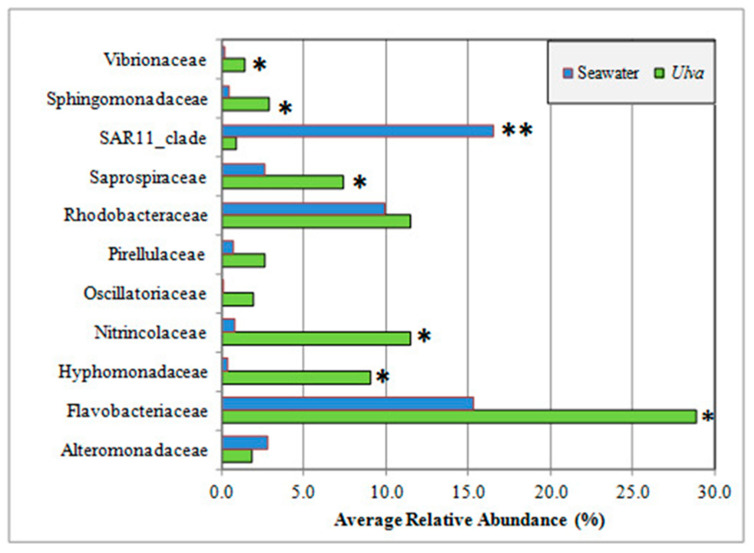
Average relative abundance of the most abundant seaweed-associated bacterial families compared with the same families identified in the surrounding seawater samples (significant one-way ANOVA values between matrices at *p*-value < 0.05 *, and *p*-value < 0.001 **.

To evaluate the potential differences between the *Ulva*-associated microbial communities and the ones present in the surrounding water column, five water samples were collected (in all the sampling sites during summer, and at SMM and PM during autumn). At the phylum level, the seaweed surface and surrounding seawater had, on average, similar community composition (Table S3), and were dominated by Proteobacteria (42.7% and 44.9%, respectively) and Bacteroidetes (36.6% and 21.2%, respectively). Considerable differences between the two microbial communities were evident at finer phylogenetic resolution (Figure 3 and Table S4). The relative abundance of seven families, out of the eleven most abundant seaweed-associated bacterial families was significantly different from the same families of the surrounding water.

### 3.4. Microbial Communities’ Indices

**Figure 4:**
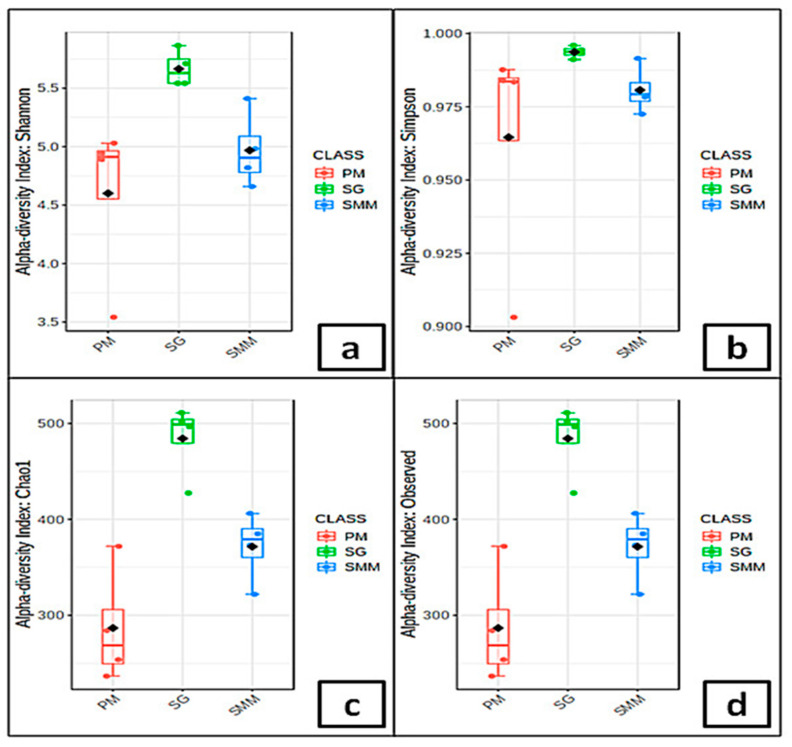
Box-plots of the bacterial α-diversity using: (**a**): Shannon Diversity Index, (**b**): Simpson’s Index, (**c**): Chao1 Diversity Index, and (**d**): observed ASVs of SAMCs among sampling periods. SMM: Santa Maria del Mare, PM: Porto Marghera, and SG: San Giuliano.

At SG, the α-diversity indexes (Figure 4a–d) of *U. laetevirens* associated bacterial communities were higher compared to the other sampling sites. Among all sampling periods except spring, PM samples exhibited the lowest α-diversity and number of distinct ASVs. The α-diversity indexes for seawater bacterial communities were approximately equal to seaweed associated ones with no significant statistical variations between the two matrices (Table S5). Conversely, the number of distinct ASVs was higher in seawater samples during summer at SMM and PM. For the Shannon index values, significant differences were shown between sites (*p* < 0.001), whereas no significant differences were detected between seasons. 

**Figure 5:**
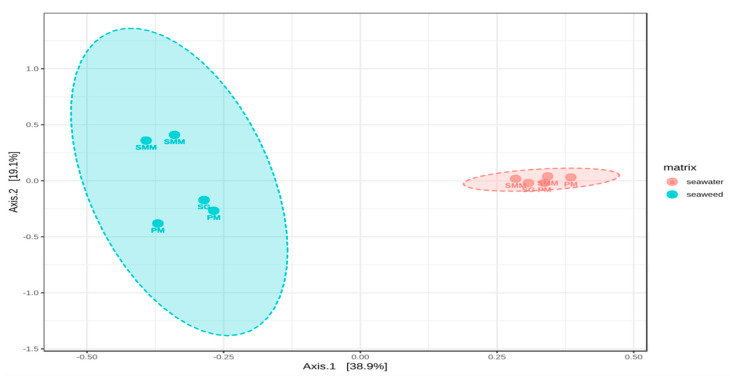
Principal coordinate analysis plot (PCoA) based on a Bray-Curtis distance matrix calculated from the square-root transformed ASV abundance data of the bacterial community on *U. laetevirens* and their surrounding seawater among sampling periods. SMM: Santa Maria del Mare, PM: Porto Marghera, and SG: San Giuliano.

The principal coordinates analysis (PCoA) performed using Bray-Curtis dissimilarity distances allowed to elucidate the variations between SAMCs among sites and their differences with the surrounding water bacterial communities. The result suggested higher variability between SAMCs sampling sites in comparison to the microbial communities of the surrounding water. Additionally, the PCoA allowed for the confirmation of the significant differences between SAMCs and the bacterial communities of the surrounding water. Figure 5 shows a clear separation along the x-axis (PC1 = 38.9% of variability) between the two matrices verified by independent clustering. This variability was also statistically assessed using the PERMANOVA test based on Bray-Curtis measures of square root transformed relative abundance of ASVs. The variation was found highly significant (Pseudo-F = 2.897, *p* < 0.005, Monte Carlo 9999 permutations).

### 3.5. Distribution of Shared and Non-Ubiquitous ASVs of SAMCs

**Figure 6:**
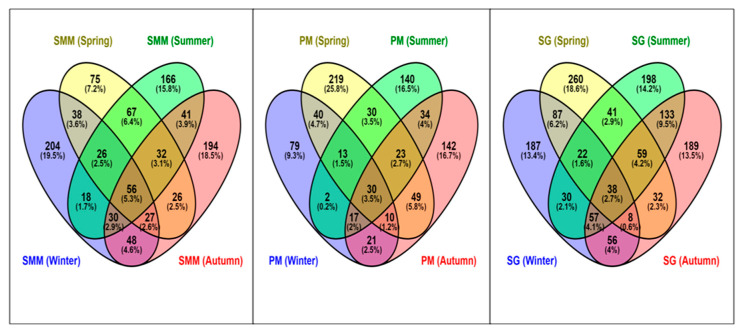
Venn diagram of ASVs from *U. laetevirens* associated bacterial community for each site of LV among sampling periods. SMM: Santa Maria del Mare, PM: Porto Marghera, and SG: San Giuliano.

The intersection among sampling periods for each site through Venn-diagrams (Figure 6) showed that PM and SG exhibited the highest percentage of non-ubiquitous ASVs during spring. The diagram showed the highest percentage of distinct ASVs in the sites during all seasons at PM (68.3 %), while the lowest percentage was found at SG (59.7 %). A total of 1241 (53.8 %) ASVs were shared among all the three sites during the sampling periods. Moreover, the highest percentage of shared (i.e., present at least in two sampling periods) ASVs was obtained at SMM with ca. 39% of the total ASVs observed in the site, while the lowest was found at PM (30%). 

### 3.6. Linear Discriminant Analysis (LDA) Effect Size (LEfSe) Characterization of SAMCs

**Figure 7:**
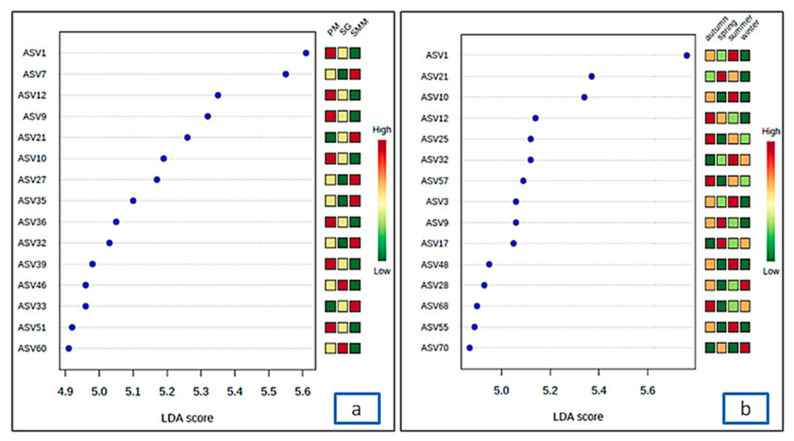
Characterization of SAMCs in different sampling sites (**a**) and sampling periods (**b**) by LEfSe analysis and LDA. Diagram of the LDA scores (log_10_) computed for the highest 15 abundant ASVs at different sites and sampling periods. SMM: Santa Maria del Mare, PM: Porto Marghera, and SG: San Giuliano.

LEfSe was applied to characterize the SAMCs among the sampling sites or sampling periods, finding 15 differentially abundant bacterial taxa with an LDA score higher than three (Figure 7). PM was characterized by a preponderance of bacterial strains belonging to the families Nitrincolaceae (ASV 1, and ASV 10) and Hyphomonadaceae (ASV 9, ASV 12, ASV 36, ASV 39, and ASV 51), that had less extent for SG and SMM (Figure 7a). LDA scores showed significant bacterial differences between sampling periods (Figure 7b). The bacterial strains of the families Flavobacteriaceae (ASV 3, ASV 25, ASV 32, ASV 57, and ASV 68) and Nitrincolaceae (ASV 1, ASV 10, and ASV 55) were predominant during the autumn and summer sampling periods. 

### 3.7. Correlation between Bacterial Communities and Environmental Parameters 

**Figure 8:**
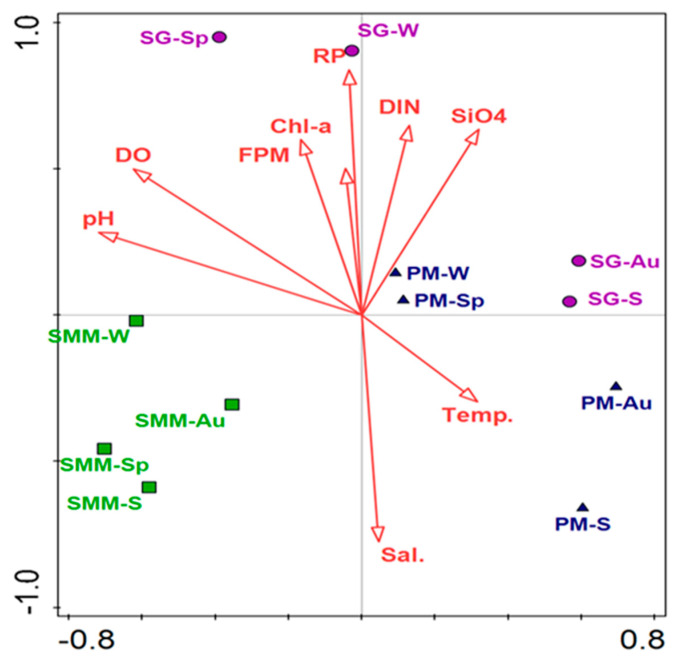
db-RDA analysis for *U. laetevirens* associated bacterial communities in response to environmental parameters. Sites: SMM = Santa Maria del Mare, PM = Porto Marghera, and SG = San Giuliano; seasons: Sp = spring, S = summer, Au = autumn, W = winter; environmental parameters: DO = dissolved oxygen, FPM = filtered particulate matter, Chl*-a* = chlorophyll*-a*, RP = reactive phosphorus; DIN = dissolved inorganic nitrogen, SiO_4_ = silicates; Temp. = temperature, Sal. = salinity.

The differences between SAMCs among the sampling periods and their correlation with environmental parameters in sampling sites were highlighted by the db-RDA analysis (Figure 8). The first two axes accounted for 36% of the total sample variance, whereas all factors accounted for 84.3% of the total variance. SMM samples were clustered together on the opposite side of the other sampling sites with slight seasonal variation among the seasons. Nutrient concentrations and the other environmental parameters were mainly associated with *U. laetevirens* bacterial communities in spring and winter in the stressed sites at PM and SG, showing a different pattern in the other seasons. The db-RDA analysis revealed that while RP (*F* = 1.482, *df* = 1, and *p* = 0.01), %DO (*F* = 1.491, *df* = 1, and *p* = 0.013) and pH (*F* = 1.547, *df* = 1, and *p* = 0.017) were significantly correlated with SAMCs, salinity (*F* = 1.178, *df* = 1, and *p* = 0.184) and water temperature (*F* = 1.157, *df* = 1, and *p* = 0.231) were not. However, SAMCs at SMM during all the sampling periods appeared to be little affected by the environmental parameters showing a slight correlation with pH, DO, and salinity. 

## 4. Discussion

This study is the first report on the characterization of the bacterial communities associated with one of the most dominant seaweed species (*U. laetevirens*) in the LV. Next-generation sequencing analysis of 16S rRNA sequences was utilized to provide a deep characterization of *U. laetevirens* microbiome across various sampling periods and different sites subjected to environmental and anthropogenic stressors. Our results clearly show that SAMCs were significantly different between the sampling sites heavily impacted by environmental and anthropogenic stressors and those less affected (Figure 8).

### 4.1. Characterization of Microbial Communities

Overall, the most abundant groups of bacteria associated with *U. laetevirens* belonged to Proteobacteria (51%) and Bacteroidetes (37%) phyla, followed by Planctomycetes and Verrucomicrobia (Figure S2 and Figure S3); in agreement with previous studies reporting the same bacterial phyla associated with marine seaweeds [14,16,48,49]. Alphaproteobacteria, followed by Bacteroidetes and Gammaproteobacteria, represented the bacterial communities’ main taxa associated with *U. intestinalis* Linnaeus [15] and *U. australis* Areschoug [49] species very close to *U. laetevirens*.

Many of the abundant *U. laetevirens* associated bacterial families (Figure 3, Table S3) that we detected (Rhodobacteraceae, Flavobacteriaceae, Hyphomonadaceae, and Pseudoalteromonadaceae) were found to be common bacteria growing in association with red, green, and brown seaweeds [50], suggesting that common properties of the seaweed holobiont may promote the growth of similar microbial taxa. For instance, Rhodobacteriaceae were described as symbionts of aquatic organisms [51]. Although not all members of the Rhodobacteraceae are considered pathogens, certain members are known to cause infections and disease in *Fucus vesiculosus* Linnaeus and *Delisea pulchra* (Greville) Montagne [52]. Also, within the Rhodobacteraceae, *Roseobacter* strains were often found in association with *U. australis* and produced a range of extracellular inhibitory compounds against common fouling organisms [53]. Besides, bacterial taxa from the genus *Aquimarina* (family Flavobacteriaceae) were previously found to be associated with marine eukaryotes’ diseases. For instance, *Aquimarina agaralytica*, which was isolated from red seaweeds, had many diverse agarases [54] that may degrade host tissue. Species of Hyphomonadaceae were previously reported as part of the microbiome of marine kelps *Nereocystis luetkeana* (K. Mertens) Postels and Ruprecht and *Macrocystis pyrifera* (Linnaeus) C. Agardh [55] with known ability to induce the normal morphogenesis of this seaweed. Bacterial taxa from the family Saprospiraceae (ca. 8.2% of the total sequences) were classified among the core microbial symbionts associated with the red seaweed *Porphyra umbilicalis* Kützing [56]. This group of bacteria may play a role in the metabolism of complex carbon resources [57].

The ASVs richness and evenness at SG, which are the highest across all sites (Figure 4), may be related to the decrease of seaweed’s physiological activity and/or antimicrobial activity. We hypothesize that the eutrophic conditions related to increased concentrations of nutrients in the water column (Table 1), and the pollutant effluents from the adjacent rivers may deteriorate the seaweed’s physiological activity. This is reflected by the increase of pathogenic species belonging to Rhodobacteraceae (*Marivita, Sulfitobacter*, and *Loktanella*), as previously observed from the aged thalli of *Cystoseira compressa* (Esper) Gerloff and Nizamuddin [58]. The in situ experiment conducted by Aires et al. [59] indicated that the addition of nutrients in *U. rigida* C. Agardh mesocosms triggered a marked shift in the microbial structure. We hypothesize that an increased concentration of nutrients may affect the richness (observed species and Chao1 indices) of bacterial community that is reflected at a finer scale/lower taxonomic levels. Furthermore, the enrichment of seaweeds surface bio-film supports the recruitment of further microbial taxa by providing potentially novel metabolic substrates and increasing microbial niche space [60]. Interestingly, the high ASVs α-diversity of SG may be correlated with chlorophyll-*a* concentrations in accordance with a previous investigation carried out by Jankowski et al. [61]. Moreover, primary productivity drivers in marine environments showed greater bacterial richness [62]. Relatively few abundant ASVs dominated the microbial community at PM, while SG hosted a more different community with the highest number of distinct ASVs and the highest percentage of non-ubiquitous ASVs (Figure 4 and Figure 6). Supporting this observation, the PM associated community was characterized by lower ASVs richness and evenness. The low evenness was due to a low number of ASVs and the dominance of ASVs, mainly belonging to the Proteobacteria that make-up nearly 58% of the sequences. We hypothesize that the lower ASVs richness found at PM resulted from anthropogenic perturbations introduced by several industrial activities affecting this area [19,30,63]. 

The composition of bacterial communities of *U. laetevirens* was different from those of the surrounding seawater (Figure 5). This agrees broadly with the findings of previous studies [12,58]. The differences between matrices may be due to the influence of the seaweed’s specific secondary metabolites, which substantially affects bacterial bio-film formation and community composition [9]. These variations were characterized by the gradual addition of ASVs belonging to the phyla Verrucomicrobia, Actinobacteria, and Cyanobacteria to seawater bacterial communities (Table S3). For instance, it was previously reported that the abundance of some members of the phylum *Verrucomicrobia* is favored by high nutrient availabilities or cyanobacterial bloom [64]. This suggests that the seaweed surfaces may be a highly selective substratum that promotes the attachment and growth of certain microbial taxa under different environmental conditions. 

Many ASVs, with a significant difference in relative abundance at the SG site were classified as belonging to the families Rhodobacteraceae and Flavobacteriaceae. In contrast, the families Hyphomonadaceae and Nitrincolaceae were significantly abundant at PM (Figure 7, Table 2). The growth of diverse bacterial groups of the Rhodobacteraceae was strongly related to the difference between the diseased and healthy seaweed *Delisea pulchra* (Greville) Montagne [65]. Among the most abundant ASVs detected in this study, two were identified as *Litorimonas* (family: Hyphomonadaceae) and *Polaribacter* (family: Flavobacteriaceae). Different species of *Litorimonas* were found in association with the green seaweed *Cladophora stimpsoni* Harvey by Nedashkovskaya et al. [66]. Similarly, *Polaribacter* was identified as marine bacteria associated with the green seaweed *U. fenestrata* Postels and Ruprecht [66]. 

### 4.2. Seasonal Variations of SAMCs

Over the sampling periods, several SAMCs exhibited significant variations. Species from the phylum Cyanobacteria were significantly abundant at PM during summer and early autumn. Eutrophication changes the aquatic environment and leads to the proliferation of cyanobacteria blooms [67]. These events are influenced by factors like temperature, pH, luminosity, and high concentrations of inorganic nutrients (nitrogen and/or phosphorus) [68]. Another important change was the spring and summer increase of Planctomycetes, mostly due to Pirellulaceae taxa’s increase. Planctomycetes were reported in association with *Macrocystis pyrifera* [12], *U. australis*, and *U. intestinalis* [15]. Planctomycetes are known for their ability to mineralize organic into inorganic compounds matching seaweeds’ nutritional requirements [69]. The summer increase of Planctomycetes is in line with the study on *Sargassum muticum* (Yendo) Fensholt [48], where the abundance of this bacterial phylum was maximum in July. 

The shift of SAMCs from winter to autumn at PM is reflected by the increase of sequences belonging to Cyanobacteria, a variation that was also observed on *Fucus vesiculosus* [15]. Likewise, the decrease of the number of ASVs and α-diversity during summer at PM (Table S5) was related to the increase of sequences belonged to Oceanospirillales which accounted for 76.7% of the total reads. Oceanospirillales prevail in the marine environment characterized by high concentrations of hydrocarbon-containing water [70,71]. We hypothesize that the remarkable changes of the environmental parameters occurring in the LV during summer, such as the significant modification of water temperature and dissolved organic matter, together with phytoplankton blooms, may lead to the dominance of few bacterial taxa, as observed in several marine microbial communities during the warmer season [72,73]. Interestingly, some of the bacterial families presented in summer samples appear to overlap with taxonomic groups associated with higher temperatures (e.g., *Vibrio*; [74]).

### 4.3. Microbial Communities and Environmental Stressors 

The global climate changes related to environmental pollution affected the SAMCs in the LV. The abiotic and biotic challenges influenced the host microbiome and resulted in a shift of SAMCs. The sampling sites experienced different environmental stressors that affected the dominance and distribution of different taxa of bacteria. The eutrophic conditions, with the consequent alteration of pH and %DO, were significantly correlated with SAMCs at SG (Figure 8). In fact, dissimilarity distance between PM and SG sampling periods indicated that interactions among microbial communities and the surrounding environmental stressors significantly affected the composition of the seaweed microbiome, consistent with previous investigations on marine seaweeds [12,58,75]. For instance, the study of Minich et al. [75] indicated that the kelp (*M. pyrifera*) microbiome was most influenced by high temperatures and a high CO_2_ partial pressure.

The increase of nutrient supply enhances the production of phytoplankton blooms and accelerates hypo-anoxic conditions and the effects of eutrophication [29]. We found a strong positive correlation between the SAMCs at SG both in spring and winter, and the high nutrient (DIN and RP) and Chl-*a* concentrations (Table 1) reflected the trophic state (Figure 8). Results are in accordance with those obtained by Jankowski et al. [61], who showed that the bacterial community composition changed and was heterogeneous within the lakes system as the trophic status increased. The concentrations of RP nitrate and %DO at SG were significantly affected by bacterial community composition (Figure 8). Dissolved oxygen was particularly correlated with denitrification. Nitrate and nitrite ammonification related genes were highly enriched on the *Macrocystis pyrifera* surface microbiome, suggesting a possible mutualistic mechanism for nitrogen cycling between the kelp and bacteria [75]. The SAMCs exhibited the highest abundance of *Alteromonas, Colwellia,* and *Pseudoalteromonas* genera (Alteromonadales order) at the sites of high DIN and RP concentrations. Indeed, Alteromonadales were perturbed by high temperatures [75]. In this study, Alteromonadales were abundant on the *U. laetevirens* surfaces during the cold period (winter and autumn) and were reduced by 71.1% in the warmer periods. Additionally, some species within the Rhizobiales order have important roles in both denitrification [76] along with nitrogen fixation in soil, and are attached to organic particles in the deep ocean and degrade xenobiotic and refractory compounds [77]. The increase in some Rhizobiales at SG would be explained by an increase in overall NO_3_^−^ and NO_2_^−^ availability in the water column, which also could be a result of bacterial metabolism.

Interestingly, the dramatic increase in water temperature under acidified conditions (low pH values) at PM resulted in a significant increase of Oceanospirillales and Vibrionales. This might be related to the possible increase of the C/N ratio in the seaweeds under low pH values. This was proved by experiments carried out by Aires et al. [78] on the seaweed *Sargassum muticum* (Yendo) Fensholt. Acidification could potentially result in shifts from healthy associated bacterial communities within seaweeds towards a higher prevalence of pathogenic bacteria and/or an increased vulnerability to disease [78]. The highest recorded pH value (8.73) at SG during spring could explain the decrease of seaweed associated Flavobacteriales (family: Flavobacteriaceae). This contrasts with a study conducted on biofilms from the Great Barrier Reef (Australia), which reported an increase in the relative abundance of Flavobacteriaceae with the decreasing pH values [79]. The univariate statistical comparison (Table S6) between sites revealed that SG was characterized by an increase of sulfate reducers (order: Desulfobacterales) and sulfur oxidizers/denitrifiers (order: Chromatiales and Campylobacterales) bacterial taxa. This may be related to the high nutrient concentrations measured at SG in comparison to the other sites. This is in line with the results of the in situ study conducted by Aires et al. [59] on the seaweeds *Ulva* and *Gracilaria*. Furthermore, the experimental investigation by Aires et al. [59] indicated that the introduction of nutrients into *Ulva* increased the abundance of bacterial species involved in sulfate reduction and organic matter decomposition (Desulfobacterales, Bacteroidales, and Clostridiales, respectively). Besides, some members of the order Campylobacterales were suggested to contribute to the nitrogen metabolism cycle for nitrate/nitrite ammonification and denitrification [80]. 

Not surprisingly, our results suggest that the SAMCs recorded at PM and SG were characterized by the presence of bacterial strains previously linked to crude oil pollution and degradation. The elevated levels of heavy metals, PAH, and pesticides, which represent a significant contamination source for both PM and SG sites [81,82], seems to stimulate the growth of Alteromonadales species (*Pseudoalteromonas, Colwellia, Aliidiomarina,* and *Alteromonas*) (4.4 and 6.8% of total sequence at PM and SG, respectively) [83]. Indeed, Alteromonodales have been found to be related to sites affected by urbanization and eutrophication [84]. Furthermore, some taxa members of Alteromonadales are metal-resistant and capable of binding copper and zinc cations, thereby reducing their toxicity [85]. Notably, in the microbial communities associated with the polychaete *Ophelina* sp., an increase of bacterial abundance from the order Alteromonadales were found in metals (copper and zinc) polluted area [86]. The toxic effect of heavy metals, and other environmental stressors, in seaweeds collected at PM and SG appears to be correlated to the overproduction of the reactive oxygen species (ROS), which triggered oxidative stress on the cell of seaweed [87]. Furthermore, species from Caulobacterales (family Hyphomonadaceae; 3.2% of the total sequences) were previously isolated from oil-contaminated environments [88], which is in line with the dominance of these bacterial taxa at PM. 

These findings suggest complex host-microbiome interaction in *U. laetevirens* at the sampling sites of LV. Indeed, the exact mechanisms of interaction have not been fully understood yet. The bacterial-dependent metabolism of stressors may modulate the host’s toxicity, while some opportunistic bacterial taxa might take advantage of the host. Besides, the high metabolic capability of bacteria may result in bacteria responding faster to external stressors. Consequently, bacterial communities should be a potential first indicator of environmental or anthropogenic stressors.

## 5. Conclusions

The exposure to various environmental and anthropogenic stressors in the LV affected the ecological system by altering the dynamics and shift of microbial communities associated with the dominant seaweed species (*U. laetevirens*). The shift of composition and dynamics of microbial communities is increasingly emphasizing the importance of seaweed-microbial interaction in response to environmental and anthropogenic changes. Further, the effect of environmental and anthropogenic stressors in the LV sampling sites was highlighted by the increased dominance and/or disappearance of bacterial taxa, which could adapt to environmental stressors or be involved in pollutant degradation. To understand why certain groups of closely related microbes are abundant on seaweeds, future research should focus on identifying the dominant symbionts’ functions to determine their importance for the host seaweed growth and adaptation.

## Figures and Tables

**Table 1:** Water column environmental parameters of LV sampling sites across sampling periods. SMM: Santa Maria del Mare, PM: Porto Marghera, and SG: San Giuliano.

Site	Season	pH	Eh (mv)	DO (%)	Sal. (PSU)	Temp. (°C)	Chl-*a* (µg/L)
**SMM**	**Winter**	8.37	298	109.9	24.9	14.2	2.08
	**Spring**	8.40	259	111.1	22.2	20.5	0.75
	**Summer**	8.32	265	110.4	32.5	25.2	0.60
	**Autumn**	8.10	312	99.3	27.0	19.0	0.75
**PM**	**Winter**	8.15	307	111.2	25.4	15.8	1.39
	**Spring**	8.33	219	118.0	23.7	25.5	1.50
	**Summer**	8.10	300	76.1	27.2	30.6	3.29
	**Autumn**	8.01	300	79.1	22.5	20.0	1.50
**SG**	**Winter**	8.12	346	94.7	6.4	14.0	2.08
	**Spring**	8.73	222	180.4	17.5	26.1	29.9
	**Summer**	8.11	570	87.0	26.5	29.8	3.59
	**Autumn**	7.91	297	76.8	26.6	19.3	1.80

(Eh: redox potential; DO: dissolved oxygen saturation; Sal: salinity; Temp: water column temperature; Chl-*a*: chlorophyll-*a* concentration).

**Table 2:** Abundance percentage and taxonomic affiliations of the 10 most abundant ASVs (amplicon sequence variant) of LV sampling sites (total of all sampling periods).

ASV	SMM	PM	SG	Phylum	Class	Order	Family	Genus
ASV1	0	6.70	0.46	Proteobacteria	Gammaproteobacteria	Oceanospirillales	Nitrincolaceae	NA
ASV7	5.35	0.54	<0.10	Bacteroidetes	Bacteroidia	Flavobacteriales	Flavobacteriaceae	NA
ASV9	0	3.06	0.53	Proteobacteria	Alphaproteobacteria	Caulobacterales	Hyphomonadaceae	Litorimonas
ASV17	1.62	1.18	0.68	Bacteroidetes	Bacteroidia	Flavobacteriales	Flavobacteriaceae	Polaribacter
ASV12	<0.10	3.20	0.27	Proteobacteria	Alphaproteobacteria	Caulobacterales	Hyphomonadaceae	Hellea
ASV5	1.47	1.32	0.64	Bacteroidetes	Bacteroidia	Flavobacteriales	Flavobacteriaceae	NA
ASV21	2.84	0	<0.10	Verrucomicrobia	Verrucomicrobiae	Verrucomicrobiales	Rubritaleaceae	NA
ASV10	0	2.52	0.16	Proteobacteria	Gammaproteobacteria	Oceanospirillales	Nitrincolaceae	NA
ASV13	0	2.36	0.15	Proteobacteria	Gammaproteobacteria	Oceanospirillales	Nitrincolaceae	NA
ASV27	2.20	0.23	<0.10	Bacteroidetes	Bacteroidia	Flavobacteriales	Flavobacteriaceae	NA

SMM: Santa Maria del Mare, PM: Porto Marghera, and SG: San Giuliano. (NA: not assigned).

## Supplementary Materials

The following are available online at https://www.mdpi.com/2076-2607/8/11/1657/s1, Table S1: Sample Metadata, Table S2: Seasonal measurement of water environmental parameters at the Lagoon of Venice, Table S3: Number of *U. laetevirens* associated bacterial sequences at phylum and class level, Table S4: Number of bacterial reads, relative abundance %, and average of the relative abundances, associated with *U. laetevirens* and in the surrounding seawater, Table S5: *α*-diversity indices of seawater and *U. laetevirens* associated microbial community at the Lagoon of Venice, Table S6: Classical univariate statistical comparison (order level) between sampling sites, Figure S1: Rarefaction curves show the observed species richness with increasing sequences depth of SAMCs, Figure S2: Seaweed associated microbial communities (SAMCs) at the phylum level across LV sampling sites, Figure S3: SAMCs at the class level across LV sampling sites.
