# Supplementary material for: Diversity and Dynamics of Seaweed Associated Microbial Communities Inhabiting the Lagoon of Venice
Source: Microorganisms. 2020 Oct 26;8(11):1657. doi: 10.3390/microorganisms8111657 (PMC7693704; doi:10.3390/microorganisms8111657)
Supplement: Supplementary file 1 [file microorganisms-08-01657-s001.zip › microorganisms-955864-SI1.pdf]

Supplementary Figures

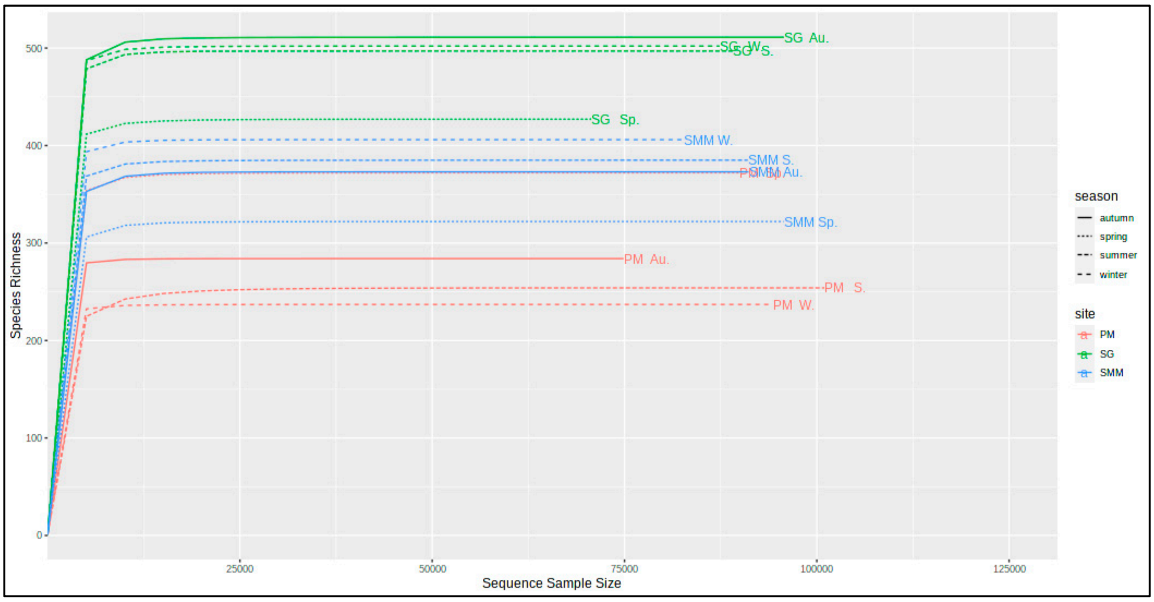

Figure S1. Rarefaction curves show the observed species richness with increasing sequences depth of SAMCs. Different colors and lines represent distinct sampling sites and seasons. SMM: Santa Maria del Mare (blue), PM: Porto Marghera (red), SG: San Giuliano (green). The curves generated by MicrobiomeAnalyst tool using 20 subsample steps and exploring all sequence reads. (**W.**: Winter, **Sp.**: Spring, **S.**: Summer, **Au.**; Autumn).

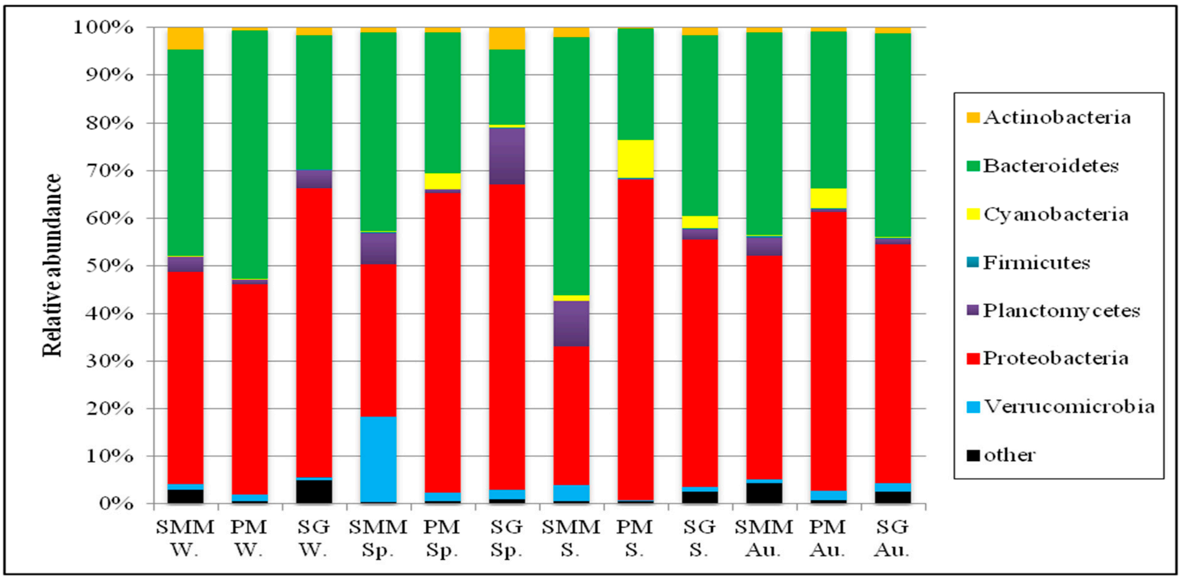

Figure S2. SAMCs at the phyla level. (**W.**: Winter, **Sp.**: Spring, **S.**: Summer, **Au.**; Autumn) across LV sampling sites. The phyla with relative abundance lower than 1.0% were grouped together. SMM: Santa Maria del Mare, PM: Porto Marghera, SG: San Giuliano.

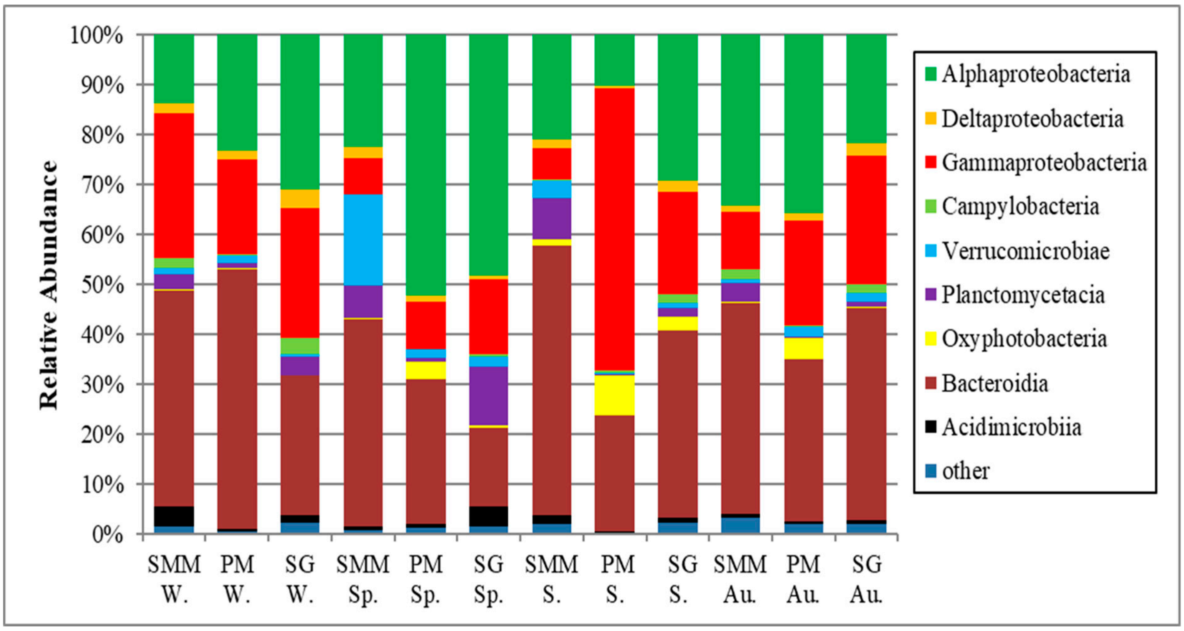

Figure S3. SAMCs at the class level. (**W.**: Winter, **Sp.**: Spring, **S.**: Summer, **Au.**; Autumn) across LV sampling sites. The classes with relative abundance lower than 1.0% were grouped together. SMM: Santa Maria del Mare, PM: Porto Marghera, SG: San Giuliano.
