# Supplementary material for: Diversity and Dynamics of Seaweed Associated Microbial Communities Inhabiting the Lagoon of Venice
Source: Microorganisms. 2020 Oct 26;8(11):1657. doi: 10.3390/microorganisms8111657 (PMC7693704; doi:10.3390/microorganisms8111657)
Supplement: Supplementary file 1 [file microorganisms-08-01657-s001.zip › microorganisms-955864-SI2.pdf]

library_ID	season	isolation_source	station_name	short_site_name
**A1**	Winter	Ulva laetevirens	Santa Maria del Mare	SMM
**A2**	Winter	Ulva laetevirens	Porto Marghera	PM
**A3**	Winter	Ulva laetevirens	San Giuliano	SG
**A4**	Spring	Ulva laetevirens	Santa Maria del Mare	SMM
**A5**	Spring	Ulva laetevirens	Porto Marghera	PM
**A6**	Spring	Ulva laetevirens	San Giuliano	SG
**A7**	Summer	Ulva laetevirens	Santa Maria del Mare	SMM
**A8**	Summer	Ulva laetevirens	Porto Marghera	PM
**A9**	Summer	Ulva laetevirens	San Giuliano	SG
**A10**	Summer	lagoon water	Santa Maria del Mare	SMM
**A11**	Summer	lagoon water	Porto Marghera	PM
**A12**	Summer	lagoon water	San Giuliano	SG
**A13**	Autumn	Ulva laetevirens	Santa Maria del Mare	SMM
**A14**	Autumn	Ulva laetevirens	Porto Marghera	PM
**A15**	Autumn	Ulva laetevirens	San Giuliano	SG
**A16**	Autumn	lagoon water	Santa Maria del Mare	SMM
**A17**	Autumn	lagoon water	Porto Marghera	PM

collection_date	geo_loc_name	lat_lon	accession	study
2016	Italy:Venice lagoon	45.325022 N 12.308514 E	SRR12433292	SRP277153
2016	Italy:Venice lagoon	45.432025 N 12.260878 E	SRR12433291	SRP277153
2016	Italy:Venice lagoon	45.470928 N 12.299428 E	SRR12433300	SRP277153
2017	Italy:Venice lagoon	45.325022 N 12.308514 E	SRR12433299	SRP277153
2017	Italy:Venice lagoon	45.432025 N 12.260878 E	SRR12433298	SRP277153
2017	Italy:Venice lagoon	45.470928 N 12.299428 E	SRR12433297	SRP277153
2017	Italy:Venice lagoon	45.325022 N 12.308514 E	SRR12433296	SRP277153
2017	Italy:Venice lagoon	45.432025 N 12.260878 E	SRR12433295	SRP277153
2017	Italy:Venice lagoon	45.470928 N 12.299428 E	SRR12433294	SRP277153
2017	Italy:Venice lagoon	45.325022 N 12.308514 E	SRR12433293	SRP277153
2017	Italy:Venice lagoon	45.432025 N 12.260878 E	SRR12433290	SRP277153
2017	Italy:Venice lagoon	45.470928 N 12.299428 E	SRR12433289	SRP277153
2017	Italy:Venice lagoon	45.325022 N 12.308514 E	SRR12433305	SRP277153
2017	Italy:Venice lagoon	45.432025 N 12.260878 E	SRR12433304	SRP277153
2017	Italy:Venice lagoon	45.470928 N 12.299428 E	SRR12433303	SRP277153
2017	Italy:Venice lagoon	45.325022 N 12.308514 E	SRR12433302	SRP277153
2017	Italy:Venice lagoon	45.432025 N 12.260878 E	SRR12433301	SRP277153

metadata

bioproject_accession	biosample_accession	title	library_strategy
PRJNA656714	SAMN15796682	16S amplicon from lagoon wate	AMPLICON
PRJNA656714	SAMN15796683	16S amplicon from lagoon wate	AMPLICON
PRJNA656714	SAMN15796684	16S amplicon from lagoon wate	AMPLICON
PRJNA656714	SAMN15796685	16S amplicon from lagoon wate	AMPLICON
PRJNA656714	SAMN15796686	16S amplicon from lagoon wate	AMPLICON
PRJNA656714	SAMN15796687	16S amplicon from lagoon wate	AMPLICON
PRJNA656714	SAMN15796688	16S amplicon from lagoon wate	AMPLICON
PRJNA656714	SAMN15796689	16S amplicon from lagoon wate	AMPLICON
PRJNA656714	SAMN15796690	16S amplicon from lagoon wate	AMPLICON
PRJNA656714	SAMN15796691	16S amplicon from lagoon wate	AMPLICON
PRJNA656714	SAMN15796692	16S amplicon from lagoon wate	AMPLICON
PRJNA656714	SAMN15796693	16S amplicon from lagoon wate	AMPLICON
PRJNA656714	SAMN15796694	16S amplicon from lagoon wate	AMPLICON
PRJNA656714	SAMN15796695	16S amplicon from lagoon wate	AMPLICON
PRJNA656714	SAMN15796696	16S amplicon from lagoon wate	AMPLICON
PRJNA656714	SAMN15796697	16S amplicon from lagoon wate	AMPLICON
PRJNA656714	SAMN15796698	16S amplicon from lagoon wate	AMPLICON

library_source	library_selection	library_layout	platform	instrument_model
METAGENOMIC	PCR	paired	ILLUMINA	NextSeq 500
METAGENOMIC	PCR	paired	ILLUMINA	NextSeq 500
METAGENOMIC	PCR	paired	ILLUMINA	NextSeq 500
METAGENOMIC	PCR	paired	ILLUMINA	NextSeq 500
METAGENOMIC	PCR	paired	ILLUMINA	NextSeq 500
METAGENOMIC	PCR	paired	ILLUMINA	NextSeq 500
METAGENOMIC	PCR	paired	ILLUMINA	NextSeq 500
METAGENOMIC	PCR	paired	ILLUMINA	NextSeq 500
METAGENOMIC	PCR	paired	ILLUMINA	NextSeq 500
METAGENOMIC	PCR	paired	ILLUMINA	NextSeq 500
METAGENOMIC	PCR	paired	ILLUMINA	NextSeq 500
METAGENOMIC	PCR	paired	ILLUMINA	NextSeq 500
METAGENOMIC	PCR	paired	ILLUMINA	NextSeq 500
METAGENOMIC	PCR	paired	ILLUMINA	NextSeq 500
METAGENOMIC	PCR	paired	ILLUMINA	NextSeq 500
METAGENOMIC	PCR	paired	ILLUMINA	NextSeq 500
METAGENOMIC	PCR	paired	ILLUMINA	NextSeq 500

design_description	filetype
Variable rDNA region V4 was amplified with universal primers and seque	fastq
Variable rDNA region V4 was amplified with universal primers and seque	fastq
Variable rDNA region V4 was amplified with universal primers and seque	fastq
Variable rDNA region V4 was amplified with universal primers and seque	fastq
Variable rDNA region V4 was amplified with universal primers and seque	fastq
Variable rDNA region V4 was amplified with universal primers and seque	fastq
Variable rDNA region V4 was amplified with universal primers and seque	fastq
Variable rDNA region V4 was amplified with universal primers and seque	fastq
Variable rDNA region V4 was amplified with universal primers and seque	fastq
Variable rDNA region V4 was amplified with universal primers and seque	fastq
Variable rDNA region V4 was amplified with universal primers and seque	fastq
Variable rDNA region V4 was amplified with universal primers and seque	fastq
Variable rDNA region V4 was amplified with universal primers and seque	fastq
Variable rDNA region V4 was amplified with universal primers and seque	fastq
Variable rDNA region V4 was amplified with universal primers and seque	fastq
Variable rDNA region V4 was amplified with universal primers and seque	fastq
Variable rDNA region V4 was amplified with universal primers and seque	fastq
