# Supplementary material for: Diversity and Dynamics of Seaweed Associated Microbial Communities Inhabiting the Lagoon of Venice
Source: Microorganisms. 2020 Oct 26;8(11):1657. doi: 10.3390/microorganisms8111657 (PMC7693704; doi:10.3390/microorganisms8111657)
Supplement: Supplementary file 1 [file microorganisms-08-01657-s001.zip › microorganisms-955864-SI3.pdf]

Seasonal measurement of environmental parameters at Lagoon of Ver

Site	Season	RP	Nitrite	Nitrate	NH_4	DIN	Silicate	Chl-a	pH
		µM	µM	µM	µM	µM	µM	µg/L	
**SMM**	**W**	0.16	0.75	5.31	0.67	6.74	7.47	2.08	8.37
	**Sp**	0.18	0.29	0.57	4.19	5.05	3.03	0.75	8.4
	**S**	0.11	0.28	4.1	2.38	6.75	9.9	0.6	8.32
	**Au**	0.16	0.48	2.62	0.57	3.67	5.89	0.75	8.1
**PM**	**W**	0.6	3.54	9.12	1.53	14.2	15.4	1.39	8.15
	**Sp**	0.76	0.93	3.42	7.4	11.7	5.69	1.5	8.33
	**S**	0.39	0.39	3.86	4.7	8.95	15.1	3.29	8.1
	**Au**	0.32	0.59	6.03	1.16	7.78	8.82	1.5	8.01
**SG**	**W**	1.4	1.91	24.3	58.5	84.7	43.7	2.08	8.12
	**Sp**	3.15	2.78	1.07	8.32	12.2	14.4	29.9	8.73
	**S**	0.81	0.58	2.92	8.64	12.1	21.7	3.59	8.11
	**Au**	0.65	2.02	14.6	10.1	26.7	19	1.8	7.91

SMM: Santa Maria del Mare, **PM**: Porto Marghera, **SG**: San Giuliano.

W.: Winter, **Sp.**: Spring, **S.**: Summer, **Au.**; Autumn

nice

Eh	DO (%)	Salinity	Temp	FPM
mv		PSU	°C	mg/L
298	109.9	24.9	14.2	18.4
259	111.1	22.2	20.5	20
265	110.4	32.5	25.2	34.8
312	99.3	27	19	42.8
307	111.2	25.4	15.8	30.8
219	118	23.7	25.5	33.4
300	76.1	27.2	30.6	31
300	79.1	22.5	20	40
346	94.7	6.4	14	20.4
222	180.4	17.5	26.1	121.5
570	87	26.5	29.8	35
297	76.8	26.6	19.3	47.8
