# Supplementary material for: Diversity and Dynamics of Seaweed Associated Microbial Communities Inhabiting the Lagoon of Venice
Source: Microorganisms. 2020 Oct 26;8(11):1657. doi: 10.3390/microorganisms8111657 (PMC7693704; doi:10.3390/microorganisms8111657)
Supplement: Supplementary file 1 [file microorganisms-08-01657-s001.zip › microorganisms-955864-SI4.pdf]

Distribution of the sequence reads for seaweed *(Ulva)* at Phylum level

**Seaweed**	winter			spring			summer	
	SMM W.	PM W.	SG W.	SMM Sp.	PM Sp.	SG Sp.	SMM S.	PM S.
Acidobacteria	13	58	634	0	93	102	16	10
Actinobacteria	4666	645	1674	1127	1072	4788	2099	259
Bacteroidetes	44330	52681	27768	42937	29556	16119	53856	24574
Chloroflexi	57	86	738	10	63	244	56	0
Cyanobacteria	178	187	10	208	3438	563	1296	8489
Deinococcus-Thermus	55	54	128	29	274	210	61	20
Epsilonbacteraeota	2120	262	3148	168	91	375	213	522
Firmicutes	94	0	139	156	24	81	23	79
Fusobacteria	427	133	181	81	0	44	101	37
Kiritimatiellaeota	120	0	0	0	0	0	54	0
Planctomycetes	3138	984	3749	6926	802	12094	9536	238
Proteobacteria	45709	44634	60234	32970	63196	65572	28960	71041
Tenericutes	0	0	0	0	0	4	0	3
Thaumarchaeota	165	21	40	39	0	0	4	0
Verrucomicrobia	1304	1478	592	18689	1827	2110	3386	225
**TOTAL**	102376	101223	99035	103340	100436	102306	99661	105497

Distribution of the sequence reads for seaweed (*Ulva*) at class level

	winter			spring			summer	
	SMM W.	PM W.	SG W.	SMM Sp.	PM Sp.	SG Sp.	SMM S.	PM S.
Acidimicrobiia	4306	584	1570	864	902	4221	1879	162
Actinobacteria	360	61	104	263	170	567	220	97
Alphaproteobacteria	13983	23536	30744	23114	52425	49373	20856	10712
Anaerolineae	57	86	738	10	63	244	56	0
Bacilli	0	0	0	156	24	32	23	0
Bacteroidia	44282	52637	27665	42937	29055	15987	53832	24538
Blastocatellia	0	0	0	0	93	0	0	0
Campylobacteria	2120	262	3148	168	91	375	213	522
Clostridia	94	0	139	0	0	49	0	79
Deinococci	55	54	128	29	274	210	61	20
Deltaproteobacteria	2008	1693	3741	2469	1374	786	1786	460
Fusobacteriia	427	133	181	81	0	44	101	37
Gammaproteobacteri	29718	19405	25749	7387	9397	15413	6318	59869
Kiritimatiellae	120	0	0	0	0	0	54	0
Mollicutes	0	0	0	0	0	4	0	3
Nitrososphaeria	165	21	40	39	0	0	4	0
Oxyphotobacteria	178	187	10	208	3438	563	1296	8489
Phycisphaerae	0	0	0	155	0	0	1316	0
Planctomycetacia	3138	984	3749	6771	802	12094	8220	238

Rhodothermia	48	44	103	0	501	132	24	36
Thermoanaerobaculia	13	58	634	0	0	102	16	10
Verrucomicrobiae	1304	1478	592	18689	1827	2110	3386	225
**TOTAL**	102376	101223	99035	103340	100436	102306	99661	105497

SMM: Santa Maria del Mare, **PM**: Porto Marghera, **SG**: San Giuliano.
W.: Winter, **Sp.**: Spring, **S.**: Summer, **Au.**; Autumn

	autumn		
SG S.	SMM Au.	PM Au.	SG Au.
194	0	368	406
1647	1035	865	1284
37764	44109	32093	43109
325	22	171	367
2576	322	4209	99
114	66	144	80
1763	2064	38	1616
163	371	174	248
118	2455	57	202
0	0	0	0
2196	3830	594	1286
51939	48873	57128	50621
34	0	10	14
0	0	39	0
965	857	1956	1777
99798	104004	97846	101109

Distribution of the sequence reads for

Seawater	Summer		
	SMM-S	PM-S	SG-S
Acidobacteria	0	0	55
Actinobacteria	2245	5141	8464
Bacteroidetes	31000	24418	23971
Chloroflexi	123	283	146
Cyanobacteria	10339	5705	12573
Deinococcus-Thermu	28	0	0
Epsilonbacteraeota	370	694	2756
Firmicutes	152	256	256
Fusobacteria	190	89	249
Kiritimatiellaeota	122	0	0
Planctomycetes	525	4936	1165
Proteobacteria	52376	51684	51655
Tenericutes	150	192	227
Thaumarchaeota	0	0	0
Verrucomicrobia	5765	7736	2786
**TOTAL**	103385	101134	104303

	autumn		
SG S.	SMM Au.	PM Au.	SG Au.
935	682	406	748
712	353	459	536
29236	35524	34891	21960
325	22	171	367
15	351	174	216
37572	44096	31829	43090
0	0	118	50
1763	2064	38	1616
148	20	0	32
114	66	144	80
2238	1485	1515	2504
118	2455	57	202
20465	11864	20722	26157
0	0	0	0
34	0	10	14
0	0	39	0
2576	322	4209	99
390	52	203	132
1806	3778	391	1154

192	13	264	19
194	0	250	356
965	857	1956	1777
99798	104004	97846	101109

the surrounding seawater at Phylum level

Autumn	
SMM-Au	PM-Au
0	15
1859	2643
34284	20464
0	0
132	66
0	0
112	110
51	86
22	78
0	0
181	470
59480	64505
95	151
0	0
9475	15802
105691	104390
