# Supplementary material for: Diversity and Dynamics of Seaweed Associated Microbial Communities Inhabiting the Lagoon of Venice
Source: Microorganisms. 2020 Oct 26;8(11):1657. doi: 10.3390/microorganisms8111657 (PMC7693704; doi:10.3390/microorganisms8111657)
Supplement: Supplementary file 1 [file microorganisms-08-01657-s001.zip › microorganisms-955864-SI5.pdf]

Distribution of the sequence reads for seaweed *(Ulva)* and the surround

	Number of reads						
	SMMs	PMs	SGs	SMMw-s	PMw-s	SGw-s	SMMau
	Ulva			seawater			Ul
AEGEAN-169_marine_gro	69	67	243	3469	2979	2163	227
Alteromonadaceae	802	2753	3480	2546	349	1033	699
Cryomorphaceae	140	60	771	4695	2589	3942	501
Cyanobiaceae	707	67	1885	10339	5665	12573	62
Flavobacteriaceae	41225	17040	26443	19534	10275	8610	34792
Halieaceae	1015	146	1475	1542	3098	4623	388
Hyphomonadaceae	4807	5283	4646	494	618	622	11470
Litoricolaceae	67	40	354	4182	3066	2687	149
Methylophilaceae	57	37	240	1042	1582	1489	142
Microbacteriaceae	207	97	623	1963	3428	6754	143
Nitrincolaceae	722	54159	4783	182	1596	2141	0
Oscillatoriaceae	27	8349	42	0	40	0	0
Pirellulaceae	7656	83	1464	417	2106	809	3708
Pseudoalteromonadacea	184	585	362	1343	146	0	227
Pseudohongiellaceae	96	0	220	1646	1375	1525	92
Puniceicoccaceae	228	59	174	2290	2482	554	323
Rhodobacteraceae	9554	4354	17049	8657	11100	17036	16122
Rubritaleaceae	2915	97	539	2075	2626	1279	385
Saprospiraceae	11672	7029	8434	1262	6363	5100	7457
SAR11_clade	213	250	1284	15054	14594	8221	1161
Sphingomonadaceae	3149	593	4768	828	769	191	4620
Vibrionaceae	1328	1462	2515	573	218	243	1797

SMM: Santa Maria del Mare, **PM**: Porto Marghera, **SG**: San Giuliano.
W.: Winter, **Sp.**: Spring, **S.**: Summer, **Au.**; Autumn

ing seawater at Family level

			% of relative abundanc					
PMau	SMMw-au	PMw-au	SMMs	PMs	SGs	SMMw-s	PMw-s	SGw-s
va	Seawater		Ulva			seawater		
368	6187	5545	0.1	0.1	0.2	3.4	2.9	2.1
1699	9148	1569	0.8	2.6	3.5	2.5	0.3	1.0
162	3899	2016	0.1	0.1	0.8	4.5	2.6	3.8
57	132	58	0.7	0.1	1.9	10.0	5.6	12.1
26408	26353	14752	41.4	16.2	26.5	18.9	10.2	8.3
364	935	1652	1.0	0.1	1.5	1.5	3.1	4.4
19166	34	76	4.8	5.0	4.7	0.5	0.6	0.6
373	2153	2833	0.1	0.0	0.4	4.0	3.0	2.6
292	2884	4457	0.1	0.0	0.2	1.0	1.6	1.4
165	1448	1642	0.2	0.1	0.6	1.9	3.4	6.5
415	22	280	0.7	51.3	4.8	0.2	1.6	2.1
1500	0	0	0.0	7.9	0.0	0.0	0.0	0.0
339	0	154	7.7	0.1	1.5	0.4	2.1	0.8
333	29	0	0.2	0.6	0.4	1.3	0.1	0.0
279	3398	4011	0.1	0.0	0.2	1.6	1.4	1.5
888	4652	8266	0.2	0.1	0.2	2.2	2.5	0.5
10963	6454	7979	9.6	4.1	17.1	8.4	11.0	16.3
715	2900	5021	2.9	0.1	0.5	2.0	2.6	1.2
3002	311	397	11.7	6.7	8.5	1.2	6.3	4.9
1611	21506	26792	0.2	0.2	1.3	14.6	14.4	7.9
1396	149	239	3.2	0.6	4.8	0.8	0.8	0.2
0	0	10	1.3	1.4	2.5	0.6	0.2	0.2

e				AVG Ulva	AVG Water
SMMau	PMau	SMMw-au	PMw-au	AVG	AVG.
Ulva		Seawater			
0.2	0.4	5.9	5.3	**0.2**	**3.9**
0.7	1.7	8.7	1.5	**1.9**	**2.8**
0.5	0.2	3.7	1.9	**0.3**	**3.3**
0.1	0.1	0.1	0.1	**0.6**	**5.6**
33.5	27.0	24.9	14.1	**28.9**	**15.3**
0.4	0.4	0.9	1.6	**0.7**	**2.3**
11.0	19.6	0.0	0.1	**9.0**	**0.4**
0.1	0.4	2.0	2.7	**0.2**	**2.9**
0.1	0.3	2.7	4.3	**0.2**	**2.2**
0.1	0.2	1.4	1.6	**0.2**	**2.9**
0.0	0.4	0.0	0.3	**11.5**	**0.8**
0.0	1.5	0.0	0.0	**1.9**	**0.0**
3.6	0.3	0.0	0.1	**2.6**	**0.7**
0.2	0.3	0.0	0.0	**0.3**	**0.3**
0.1	0.3	3.2	3.8	**0.1**	**2.3**
0.3	0.9	4.4	7.9	**0.3**	**3.5**
15.5	11.2	6.1	7.6	**11.5**	**9.9**
0.4	0.7	2.7	4.8	**0.9**	**2.7**
7.2	3.1	0.3	0.4	**7.4**	**2.6**
1.1	1.6	20.3	25.7	**0.9**	**16.6**
4.4	1.4	0.1	0.2	**2.9**	**0.4**
1.7	0.0	0.0	0.0	**1.4**	**0.2**
