# Supplementary material for: Diversity and Dynamics of Seaweed Associated Microbial Communities Inhabiting the Lagoon of Venice
Source: Microorganisms. 2020 Oct 26;8(11):1657. doi: 10.3390/microorganisms8111657 (PMC7693704; doi:10.3390/microorganisms8111657)
Supplement: Supplementary file 1 [file microorganisms-08-01657-s001.zip › microorganisms-955864-SI6.pdf]

Alpha diversity indices of seawater and *U. laetevirens* associated mic

			Alpha diversity indix			
**Sample**	**Matrix**	**Season**	**Chao1**	**Shannon**	**Simpson**	**Observed**
**SMM**	***Ulva***	Summer	307	4.63	0.969	307
**PM**			237	3.47	0.900	237
**SG**			439	5.57	0.994	439
**SMM**		Autumn	298	4.47	0.972	298
**PM**			234	4.70	0.979	234
**SMM**	**Seawater**	Summer	410	5.31	0.990	410
**PM**			354	5.22	0.988	354
**SG**			232	4.96	0.988	232
**SMM**		Autumn	330	4.88	0.983	330
**PM**			378	4.98	0.981	378

* **SMM**: Santa Maria del Mare, **PM**: Porto Marghera, **SG**: San Giuliano.

* **W.**: Winter, **Sp.**: Spring, **S.**: Summer, **Au.**; Autumn

crobial community at Lagoon of Venice.
