# Supplementary material for: Diversity and Dynamics of Seaweed Associated Microbial Communities Inhabiting the Lagoon of Venice
Source: Microorganisms. 2020 Oct 26;8(11):1657. doi: 10.3390/microorganisms8111657 (PMC7693704; doi:10.3390/microorganisms8111657)
Supplement: Supplementary file 1 [file microorganisms-08-01657-s001.zip › microorganisms-955864-SI7.pdf]

Classical Univariate Statistical Comparison (Order level) between sites

Order	P-value	FDR	F Statistics	Site
Desulfuromonadales	0.0010	0.04559	16.555	SG
Bacteroidales	0.0015	0.04559	14.603	SG
Steroidobacterales	0.0077	0.15015	8.7542	SG
Myxococcales	0.0117	0.15015	7.6019	SG
Desulfobacterales	0.0123	0.15015	7.4569	SG
Gammaproteobacteria_Incert	0.0203	0.1507	6.1943	SG
Anaerolineales	0.0208	0.1507	6.1423	SG
Deinococcales	0.0216	0.1507	6.0506	SG
Thermoanaerobaculales	0.0243	0.1507	5.7775	SG
Thiomicrospirales	0.0247	0.1507	5.7417	SG
Pirellulales	0.0286	0.15858	5.4142	SMM
Campylobacterales	0.0347	0.17425	4.9943	SG
Oxyphotobacteria_Incertae_S	0.0371	0.17425	4.8548	SMM

FDR: False discovery rate

SMM: Santa Maria del Mare, **PM**: Porto Marghera, **SG**: San Giuliano.
